# Conservative management of patients with endometrial intraepithelial neoplasia (EIN): Factors that could affect response and pregnancy rates

**DOI:** 10.55730/1300-0144.5384

**Published:** 2021-09-30

**Authors:** Christos IAVAZZO, Ioannis GKEGKES

**Affiliations:** 1 Department of Gynaecological Oncology, Metaxa Cancer Hospital, Piraeus, Greece; 2 Athens Colorectal Laboratory, Athens, Greece; 3 Department of Colorectal Surgery, Royal Devon and Exeter NHS Foundation Trust, Exeter, UK

Dear Editor,

With a great deal of interest, we read your article entitled “Outcomes of the conservative management of the patients with endometrial intraepithelial neoplasia/endometrial cancer: Wait or treat! by Işçi Bostanci et al. [1].

This is a retrospective study presenting the conservative management of 38 patients with either endometrial cancer (EC) (6/38) or endometrial intraepithelial neoplasia (EIN) (31/38) with progestins (such as medroxyprogesterone acetate, megestrol acetate, or levonorgestrel-releasing intrauterine device) and follow-up endometrial biopsies every 3–6 months.

We would like to focus on the results of the 32 patients with EIN. The majority of them (28/32–87.5%) had a response while 8 (25%) had a relapse and 4 (12.5%) had persistent disease. Seven pregnancies were achieved with five live births, and all of the pregnancies were seen in the EIN group [1]. One patient with EIN progressed to advanced endometrioid carcinoma [1].

Recently, Cordeiro Mitchell et al. published a similar retrospective study, which revealed that among 54 patients who were conservatively treated for EC/EIN, nearly 20% developed intrauterine synechiae [2]. We would like to ask the authors if they can provide us with the intrauterine synechiae rates in their retrospective study and inform us whether there might be a correlation with the lower pregnancy rates in their series.

Another group showed the addition of metformin to progestin therapy could have little impact on response rates and lower live-birth rates [3]. We would like to ask the authors if they have any experience with the combination of metformin and progestin in their cohort.

Finally, Rafone et al. in a systematic review and meta-analysis stated that diabetes mellitus does not affect the responsiveness of such pathology to conservative treatment [4]. As the BMI of the Turkish patients is quite high, we would like to ask the authors to further inform us if they found any correlation with diabetes mellitus either for disease progression or fertility outcome.

Once again, we would like to thank the authors for their excellent work, and we would appreciate it if they could present any of these requested data if they are available.
